# Low Molecular Weight Volatile Organic Compounds Indicate Grazing by the Marine Rotifer *Brachionus plicatilis* on the Microalgae *Microchloropsis salina*

**DOI:** 10.3390/metabo10090361

**Published:** 2020-09-04

**Authors:** Carolyn L. Fisher, Pamela D. Lane, Marion Russell, Randy Maddalena, Todd W. Lane

**Affiliations:** 1 Bioresources and Environmental Security Department, Sandia National Laboratories, P.O. Box 969, Livermore, CA 94551, USA; clfish@sandia.gov; 2 Systems Biology Department, Sandia National Laboratories, P.O. Box 969, Livermore, CA 94551, USA; plane@sandia.gov; 3 Energy Analysis and Environmental Impacts Division, Lawrence Berkeley National Laboratory, 1 Cyclotron Rd., Berkeley, CA 94720, USA; mlrussell@lbl.gov (M.R.); rlmaddalena@lbl.gov (R.M.)

**Keywords:** *Microchloropsis salina*, *Brachionus plicatilis*, volatile organic compounds, biomarkers, headspace sampling, pond crash

## Abstract

Microalgae produce specific chemicals indicative of stress and/or death. The aim of this study was to perform non-destructive monitoring of algal culture systems, in the presence and absence of grazers, to identify potential biomarkers of incipient pond crashes. Here, we report ten volatile organic compounds (VOCs) that are robustly generated by the marine alga, *Microchloropsis salina*, in the presence and/or absence of the marine grazer, *Brachionus plicatilis*. We cultured *M. salina* with and without *B. plicatilis* and collected in situ volatile headspace samples using thermal desorption tubes over the course of several days. Data from four experiments were aggregated, deconvoluted, and chromatographically aligned to determine VOCs with tentative identifications made via mass spectral library matching. VOCs generated by algae in the presence of actively grazing rotifers were confirmed via pure analytical standards to be pentane, 3-pentanone, 3-methylhexane, and 2-methylfuran. Six other VOCs were less specifically associated with grazing but were still commonly observed between the four replicate experiments. Through this work, we identified four biomarkers of rotifer grazing that indicate algal stress/death. This will aid machine learning algorithms to chemically define and diagnose algal mass production cultures and save algae cultures from imminent crash to make biofuel an alternative energy possibility.

## 1. Introduction

Volatile organic compounds (VOCs) have been characterized as abundant, influential, and chemically-diverse natural products generated by a variety of biological systems. Terrestrial systems are known to produce VOCs for a variety of functions and mechanisms, including host defense, immune system stimulation, attractants for pollinators, interkingdom signaling, and intra- and inter-species communication [1,2]. Real-time monitoring of plant volatilomics systems will further advance chemical discoveries [3]. The breadth of VOCs in aquatic systems, and specifically marine systems, and their ecological roles, by comparison, are not as well understood. In the absence of an algal bloom, microalgae present a unique analytical challenge for VOC discovery due to low natural aquatic concentrations of algal cells, high-salinity growth conditions, and integrated growth and dependence of microalgae on other microbial systems [4]. Algal mass production systems provide an opportunity to study in vivo VOC production without being limited to the study of algal blooms.

Industrial-scale microalgae production systems have the potential to supply a renewable source of biofuel, high value products, and feedstocks to the world [5]. However, microalgae production often carries high capital and operational costs, which are barriers to commercialization especially under conditions of low algal biomass productivity due to abiotic environmental stressors, infection and disease, or competition from invasive species or strains [6]. Algal cultivation systems are susceptible to biotic contaminants, resulting in an estimated 30% loss of the annual algal crop production per year [7,8]. Current algal crop protection strategies include prophylactic treatment with biocides [9] and increasing the CO_2_ levels within the culture to inhibit heterotrophic contaminants [10]. These strategies can lead to desensitization of target organisms, detrimental effects on the environment, and unsustainable costs for the algal industry. A much more sustainable and economical approach for mass algal production is to only treat algal cultures with chemicals when a biological threat is detected [11]. Currently, the most common method for monitoring algal ponds involves microscopy, which is laborious, requires a skilled workforce, and may lack sufficient sensitivity for timely detection of and treatment for biological contaminants [12]. Several molecular techniques and automated imaging flow cytometry systems are under development, but additional, potentially low-cost and high-throughput methods are needed [11]. Early detection of biological stress through non-invasive, in vivo VOC detection can aid production specialists by providing crucial time to apply one or several treatments to save an algal production system from complete collapse.

Most of the previous work for analyzing VOCs from microalgae involves concentration and destruction of the algal biomass or purge and trap sampling methods to generate detectable chemical signals from the algae (for review, see [13]). The most commonly used method for sampling VOCs from algal systems is to harvest and concentrate the algal biomass, then heat or solvent extract the pellet and collect headspace from these samples. For example, four marine algal strains (*Botryococcus braunii*, *Rhodomonas* species, *Tetraselmis* species, and *Nannochloropsis oculate*) and one freshwater microalga (*Chlorella vulgaris*) were freeze dried and heated to 40 °C in order to generate VOCs prior to sample collection via solid-phase microextraction fibers (SPMEs) [14]. Similarly, freeze-dried pellets of six algae (*Nitzschia closterium*, *Chaetoceros calcitrans*, *T. weissflogii*, *Platymonas helgolandica*, *Nannochloropsis* sp., and *Dicrateria inornata*), taken at different growth phases, were heated to 40 °C to produce volatiles [15]. Although these methods of VOC sampling in algae systems are in common use, they fail to supply in vivo or in situ chemical information for the algal culture. Furthermore, these studies might inaccurately suggest VOCs generated by living or stressed algal cultures due to VOC generation and sampling methodologies that rely on centrifuging, heating, or otherwise agitating the algal cells to produce a detectable chemical signal. Very few studies have focused on in vivo headspace sampling of algal cultures and even fewer have assessed VOCs generated by algae in the presence of a biotic stressor (for a more comprehensive review of algal VOC sampling, production, analysis, see [4]). Our methods are more similar to the purge and trap methods in which cultures are sparged with a stripping gas and the volatiles are concentrated from the gas stream on a sorbent trap prior to elution and analysis. This method has been used for the detection of isoprene and other monoterpenes generated by four algal strains (*Thalassiosira weissflogii*, *Thalassiosira pseudonana*, *Pleurochrysis carterae*, and *Rhodomonas salina*) after being subjected to four hours of light stress conditions [16]. The system used for this work, described by Reese et al. (2019), is a simplified version of a purge and trap system using the normal aeration of the algal culture to provide stripping gas and thermal desorption tubes as a trap [17]. This sampling system has the advantage of being adaptable to all culture scales, from the laboratory to algal production systems of 100,000 L or more. 

Recently, Reese et al. (2019) reported results from the first in situ analysis of VOCs generated during grazing by the marine rotifer, *Brachionus plicatilis*, on marine microalgal cultures of *Microchloropsis salina* [17]. For this work, solid-phase microextraction fibers with polydivinylbenzene (SPME-PDVB) sorbent material was used for passively sampling headspace VOCs. This published work identified a number of putative carotenoid oxidation products as potential indicator molecules of grazing by *B. plicatilis* of *M. salina* [17]. During these same experiments, multi-sorbent adsorption tubes containing Carbopack B and X were also used to actively collect headspace samples. Carbopack-B/X™ and SPME-PDVB have different chemical affinities and both methods perform well in humid environments [18,19]. The Carbopack-B/X^TM^ active sampling uses known sample volumes and sparging rates that produce a dynamic flow through headspace so that quantitative VOC data can be reported [20,21]. Thus, the sampling approaches provide two separate but complementary VOC datasets from the same set of experiments. Our previously reported results focused on the analysis of higher molecular weight compounds, in particular the role of carotenoid oxidation products, as potential indicators of rotifer grazing. In order to maintain this focus of the previous study and to simplify the discussion thereof, it was decided to separate the results from SPME and Carbopack analysis into two separate reports. As we describe below, the Carbopack analysis resulted in the identification of a broadly different set of lower molecular weight compounds with little overlap with the previously reported results [17]. In addition, we also report the results from the analysis of an additional time course experiment, and the confirmation and quantitation of chemical identifications not included in our previous work. 

Here, we summarize the chemical signals captured by the Carbopack B/X™ sorption tube during these experiments. We analyzed the Carbopack B/X™ sorption tubes via thermal desorption gas chromatography–mass spectrometry (TD-GC/MS) and tentatively identified and quantified 10 differentially-produced metabolites generated by algae and/or algae actively undergoing grazing by marine rotifers. We then determined which metabolites would be the most useful for differentiating healthy from unhealthy mass cultures of algae. Our data can contribute to a larger “chemical library” to better understand the diversity of metabolites generated by microalgae in vivo in order to provide an early diagnostic for algal culture systems that are undergoing stress, grazing or other physical disruption.

## 2. Results and Discussion

### 2.1. Headspace Sampling with Carbopack B/X™ Sorption Tubes

**Figure 1:**
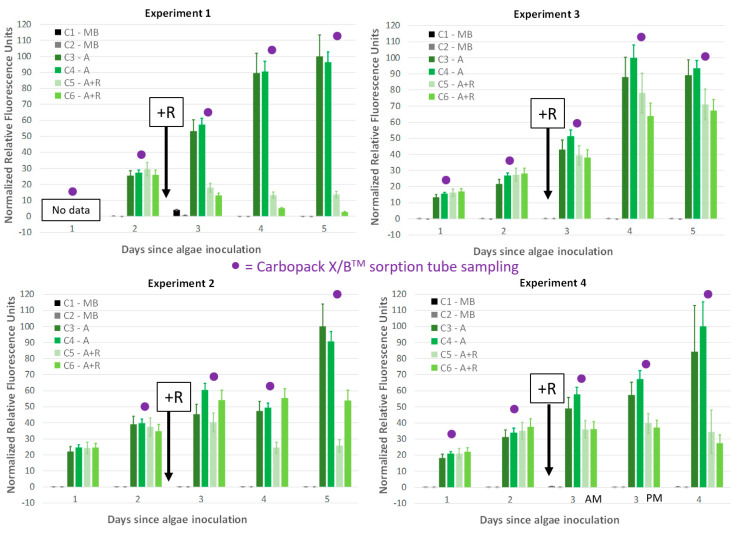
Daily chlorophyll fluorescence time points tracked algal concentrations for each of the four experiments. Carboys 1 and 2 (C1, C2) were media blanks (MB) and Carboys 3, 4, 5, and 6 (C3, C4, C5, C6) were inoculated with algae (A) at the beginning of the time course (day 0, fluorescence data not shown) and only C5 and C6 had rotifers added (+R) after 48 h of algal cultivation (A + R). Carbopack B/X™ sorption tube sampling occurred at each of the time points denoted with a purple dot (•) on each graph. Data for Experiments 1–3 derived from [17]. Error bars represent standard deviation taken from duplicate chlorophyll fluorescence measurements for each culture.

Active headspace sampling of algal mass cultures with Carbopack B/X™ sorption tubes was performed as an orthogonal detection method to the SPME analysis of the same algal mass culture experiments previously published in [17]. The 15 L *Microchloropsis salina* cultures were monitored daily via chlorophyll fluorescence before and after addition of the marine rotifer, *B. plicatilis* (Figure 1). As previously described [17], rotifers (R) were added to algal (A) cultures in carboys 5 and 6 (C5, C6) at 88 R mL^−1^ after 48 h of algal growth in carboys 3–6 (C3–C6). Despite maintaining consistent mass culture conditions for all four experiments, the algal crash rate by the grazing rotifers was highly variable, with Experiment 1 showing almost a 70% reduction in algal biomass after 24 h of rotifer grazing while Experiment 3 showed only slight difference in fluorescence between algae only (A) and algae with rotifer (A + R) cultures after 24 h of grazing. It is likely that this large difference in rotifer grazing rate is due to the different lots of rotifers purchased and used for each experiment. Although all purchases of marine rotifers were made from the same vendor (Reed Aquaculture) at the same density and rotifers were washed, concentrated, and aliquoted in the same way, biological differences between the rotifers purchased due to differences in their physiological state and/or environment likely contributed to the different grazing rate exhibited by each of the four experiments in Figure 1.

However, despite these observed experimental differences in rotifer grazing rates, there was consistency between chemical biomarkers generated by algae in the presence and absence of rotifer grazing. Across all 108 Carbopack B/X™ sorption tubes samples, collected from all four experiments, mass spectral deconvolution and chromatographic alignment identified the presence of 1266 individual chemical species. From this, the chemical signals were mathematically scaled according to sampling times and then tentatively identified by reference to the National Institutes of Standards and Technology 2014 (NIST14) chemical library database. The resulting data were rigorously filtered to down select for chemical signals that fit three conditions: (1) most intense peak in the mass spectra (i.e., base peak) present at an abundance of 10-fold the average of travel and media blank background signals (ratio of 1:10); (2) present in more than one biological replicate (A and/or A + R) within an experiment; and (3) present in more than one experiment. Of 1266 chemical species, only 60 compounds survived these three conservative filtering parameters. The mass-to-charge ratio (*m*/*z*) of the base peak in the mass spectra, retention time (RT), and NIST14 confidence level of the library match for the mass spectra for each of the 10 chemicals, found to be “robustly” present in at least three or more biological replicates within an experiment *and* present in at least three or more experiments, are summarized in Table 1. The remaining 50 chemicals are summarized in Supplementary Table S1. The chemicals summarized in Table 1 were further evaluated as biomarkers indicative of algal health. Even though the same experiments as in Reese et al. [17] were analyzed for this work, radically different VOC markers for grazing are summarized herein due to the different sampling collection using Carbopack B/X^TM^ sorption tubes.

### 2.2. Compound ***1***

**Figure 2:**
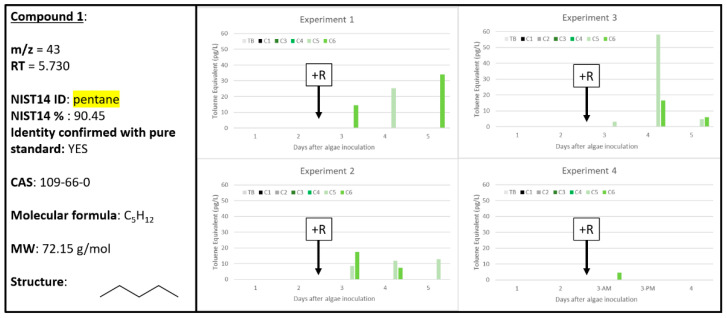
Compound **1** confirmed to be pentane (CAS: 109-66-0). Graphs indicate quantity of Compound **1** reported in each of the four experiments in pg L^−1^, where Carboys 1 and 2 (C1, C2) were media blanks (MB) and Carboys 3, 4, 5, and 6 (C3, C4, C5, C6) were inoculated with algae (A) at the beginning of the time course (day 0, data not shown) and only C5 and C6 had rotifers added (+R) after 48 h of algal cultivation (A + R).

Compound **1**, identified as pentane by the NIST14 database at 90.45% and confirmed using pure standard, is one of the lowest molecular weight (MW) chemical signals captured by the Carbopack B/X™ sorption tubes (72.15 g mol^−1^ MW). Compound **1** was determined to be correlated to A + R samples, only, in all four of the experiments (Figure 2) and was highest after 48 h of rotifer grazing in Experiment 3 at almost 60 pg L^−1^. For all other samples for which Compound **1** was detected, it was typically at approximately 20 to 30 pg L^−1^. Pentane is an observed product of lipid catabolism by hydroperoxide lyase, known to act on linoleic acid in some algae, such as the green alga *Chlorella pyrenoidosa* [22] and the blue-green alga *Oscillatoria* sp. [23]. The authors of these works concluded that there are no known roles for these metabolites in either algal species, but it is possible that fatty acid breakdown could have an unknown allelopathic effect for grazer deterrence. Here, our data support that pentane is a reliable biomarker for algal grazing as indicated by the robust observation and correlation to A + R samples in Experiments 1–3 (Figure 2).

### 2.3. Compound ***2***

**Figure 3:**
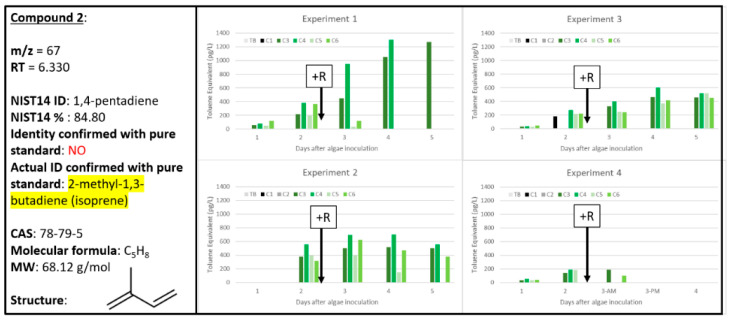
Compound **2** confirmed to be 2-methyl-1,3-butadiene (CAS: 78-79-5). Graphs indicate quantity of Compound **2** reported in each of the four experiments in pg L^−1^, where Carboys 1 and 2 (C1, C2) were media blanks (MB) and Carboys 3, 4, 5, and 6 (C3, C4, C5, C6) were inoculated with algae (A) at the beginning of the time course (day 0, data not shown) and only C5 and C6 had rotifers added (+R) after 48 h of algal cultivation (A + R).

Compound **2**, tentatively identified as 1,4-pentadiene by the NIST14 database at 84.8% (Figure 3), was the smallest chemical (MW = 68.12 g mol^−1^) to be correlated to the biological samples in all four experiments. Interestingly, there is not a clear pattern for Compound **2** in terms of correlation to A or A + R samples. In Experiment 1, where only 30 min of sampling was employed for sample collection, Compound **2** was correlated with healthy algal cultures with increasing abundance over time as algal concentration increased in C3 and C4. The Compound **2** signal diminished quickly within 24 h after rotifer addition (C5, C6). Similarly, in Experiments 2 and 4, the Compound **2** signal diminished over time with decreased algal concentrations in C5 and C6 with active rotifer grazing. Experiment 3, however, showed very different results for Compound **2** abundance over the time course of the experiment: signal increased daily and at similar levels across all biological samples (C3–C6). This could be explained by Experiment 3 displaying the lowest impact of grazing rotifers of the four experiments. Experiment 3 exhibited approximately a 30% decrease in algal concentration between A and A + R cultures by day 5, compared to 50–90% decreases in Experiments 1, 2, and 4 (Figure 1). If Compound **2** is indeed a signal generated by healthy algae, then perhaps this high relative concentration of healthy algae still present within the A + R cultures in Experiment 3 resulted in the higher relative signal. Also interesting, Experiment 4 indicated the lowest levels of Compound **2** (in all samples), despite employing the longest sampling time of 120 min. The low levels could be related to sample breakthrough for Compound **2** on the Carbopack B/X™ matrix. Regardless, with 200 to almost 1400 pg L^−1^ of Compound **2** collected in Experiments 1–3, it was the highest quantity of VOC collected over the four experiments.

Additionally, there was another metabolite (Compound **S2**) tentatively identified as 1,4-pentadiene that was less robustly associated with A and/or A + R cultures, but still nonetheless met the threshold for inclusion in Supplementary Table S1. Compound **S2** was excluded from Table 1 because it only was observed in two experiments and insufficient replicates. However, Compound **S2** was exclusively found in biological samples of A + R, indicating it might be a weak chemical signal associated with rotifer grazing. The extracted chromatograms for Compound **2** and Compound **S2** are similar with the exception that considerably less ions were observed for Compound **S2** (Supplementary Figure S1). Notably, Compound **2** and **S2** both have different retention times—6.330 and 7.525 min, respectively—and thus could not both be the same 1,4-pentadiene chemical signal. It is possible that *cis/trans* or E/Z isomers could explain this difference in retention time, yet the same reported chemical signal. It is certainly possible that 1,4-pentadiene is generated by the microalgal cultures as it has been previously reported as an algal metabolite. For example, upon ethenolysis of unsaturated fatty acids within oil from *Phaeodactylym triconutum*, 1,4-pentadiene is produced [24]. While it is unknown if *Microchloropsis salina* is capable of this same degradation reaction, there is absolutely an abundance of precursor polyunsaturated fatty acids (PUFAs) present within *M. salina* that would make 1,4-pentadiene a plausible metabolic byproduct. 

When a pure standard of 1,4-pentadiene was analyzed, it did not confirm that Compound **2** nor Compound **S2** was 1,4-pentadiene. The NIST14 electron ionization mass spectrometry data for 1,4-pentadiene look similar to that of 2-methyl-1,3-butadiene, also known as ‘isoprene’ (Supplementary Figure S2). Comparisons of the extracted ion chromatograms for Compound **2** and Compound **S2** with the NIST14 reference for 1,4-pentadiene and isoprene (Supplementary Figure S3) further support that either chemical assignment, or another structural isomer, is certainly plausible. Upon analysis of a pure standard of isoprene, Compound **2** was indeed confirmed to be 2-methyl-1,3-butadiene, or ‘isoprene’. There is extensive past work characterizing isoprene as an important VOC of plants and algae. Rivaling the abundance of atmospheric methane, isoprene contributes to approximately one-third of the extensive hydrocarbon load released naturally by vegetation and oligotrophic water systems worldwide [25], especially highest during warm months with excessive heat and light [26]. Isoprene is implicated in ozone creation and lower cellular oxidative stress through reacting with reactive oxygen species (ROS). Currently, the best evidence for why plants emit isoprene is heat tolerance. Similarly, cyanobacteria, diatoms, and green algae have shown increased emission of isoprene as a result of light stress [13]. In plants, isoprene and more complex terpenes have been found to have important roles as antioxidant agents as they will scavenge ROS under various abiotic stresses, such as heat, light, and drought. It is believed that algae also utilize isoprene to lower oxidative stress within cells. Based on this prior work, it is possible that *M. salina* is generating isoprene to similarly scavenge ROS and lower oxidative stress in high density algal cultures or cultures experiencing algal grazing. However, because Compound **2** does not specifically correlate with healthy algae or algae experiencing rotifer grazing, these data do not support the use of isoprene as a useful biomarker to inform on algal health.

### 2.4. Compounds ***3*** and ***4***

**Figure 4:**
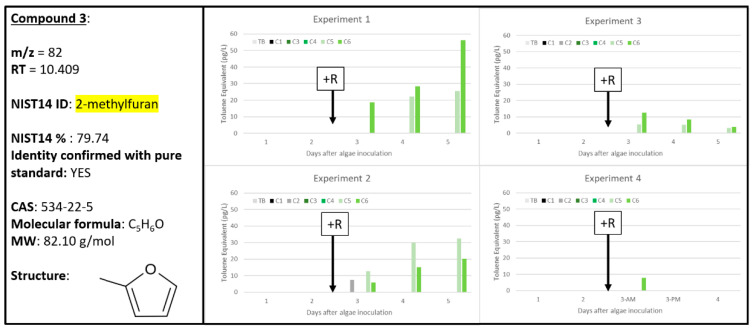
Compound **3** confirmed to be 2-methylfuran (534-22-5). Graphs indicate quantity of Compound **3** reported in each of the four experiments in pg L^−1^, where Carboys 1 and 2 (C1, C2) were media blanks (MB) and Carboys 3, 4, 5, and 6 (C3, C4, C5, C6) were inoculated with algae (A) at the beginning of the time course (day 0, data not shown) and only C5 and C6 had rotifers added (+R) after 48 h of algal cultivation (A + R).

**Figure 5:**
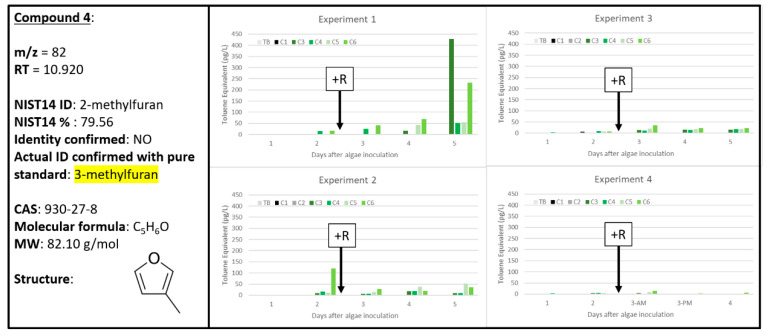
Compound **4** confirmed to be 3-methylfuran (930-27-8). Graphs indicate quantity of Compound **4** reported in each of the four experiments in pg L^−1^, where Carboys 1 and 2 (C1, C2) were media blanks (MB) and Carboys 3, 4, 5, and 6 (C3, C4, C5, C6) were inoculated with algae (A) at the beginning of the time course (day 0, data not shown) and only C5 and C6 had rotifers added (+R) after 48 h of algal cultivation (A + R).

Based on their similar extracted chromatograms (Supplementary Figure S4), both Compounds **3** and 4 are very similar chemical structures (likely both furans) and both align well with the NIST14 reference for 2-methylfuran (Supplementary Figure S5). However, Compounds **3** and 4 clearly cannot be the same chemical signal due to their different retention times (10.409 and 10.920 min, respectively) and vastly different chemical response rates over the four experiments: Compound **3** has a high correlation to A + R samples only (Figure 4) while Compound **4** was found in both A and A + R samples, with less of recognizable pattern (Figure 5). Interestingly, Compound **3** increased over time for A + R samples in Experiments 1 and 2 but decreased over time in Experiment 3 and is only present in one A + R sample in Experiment 4. Experiments 2 and 3 were both sampled for 60 min each, but Experiment 3 did have the lowest grazing rate, as discussed previously (Figure 1). Compound **4** has less of an obvious pattern, when compared to Compound **3**, as it was found in A and A + R samples throughout all four experiments. On day 5, C3 containing only algae exhibited the greatest abundance of Compound **4** at 450 pg L^−1^ (Experiment 1, Figure 5). However, in the same Experiment 1 dataset, Compound **4** was also found at almost 250 pg L^−1^ in C6, an A + R sample with extremely depleted algal concentration (Figure 1).

This observed ambiguity in signal intensity between biological samples and experiments does not support use of Compound **4** as a reliable biomarker that can distinguish between algal health or algal stress/death. However, since Compound **3** presence clearly tracks with A + R samples, this makes Compound **3** a useful biomarker of algal stress/death. A pure standard of 2-methylfuran was analyzed and confirmed as the identity of Compound **3** (Figure 4) and similarly, 3-methylfuran was confirmed to be Compound **4** (Figure 5). There is precedence for 2-methylfuran in the literature as a plant molecule and allelochemical. Connick et al. (1989) found that Amaranth residues, of the *Amaranthus* genus consisting of herbs, grains, and rice plants, generate 2-methylfuran with significant inhibition of germination of carrot, potato, and onion seedlings when compared to control samples [27]. Additionally, 2-methylfuran was identified as one of three alkylfurans (2-methylfuran, 3-ethylfuran, and 2-pentylfuran) found in the green alga *Capsosiphon fulvescens* [28]. Previously, 3-methylfuran has been identified as a volatile generated from various fungal species and could potentially be a biomarker for mold [29]. Furans, especially 2- and 3-methylfuran, have been identified as oxidation products of PUFAs, such as linolenic acid, and thus have been identified as food contaminants [30]. It is also possible that oxidation of isoprene resulted in the formation of the 2- and/or 3-methylfuran chemical signals [31]. This previous work supports that 2-methylfuran and 3-methylfuran are likely oxidation products generated by algae.

### 2.5. Compound ***5***

**Figure 6:**
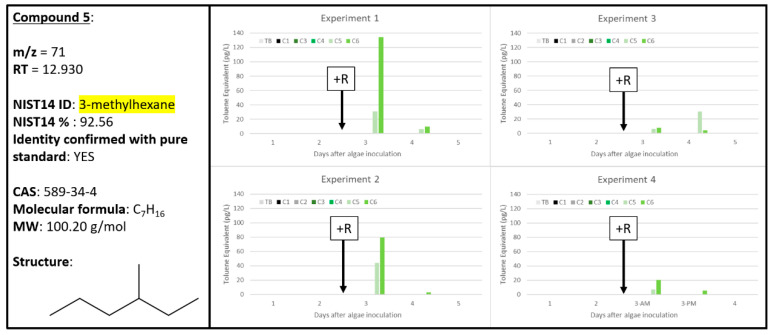
Compound **5** confirmed to be 3-methylhexane (589-34-4). Graphs indicate quantity of Compound **5** reported in each of the four experiments in pg L^−1^, where Carboys 1 and 2 (C1, C2) were media blanks (MB) and Carboys 3, 4, 5, and 6 (C3, C4, C5, C6) were inoculated with algae (A) at the beginning of the time course (day 0, data not shown) and only C5 and C6 had rotifers added (+R) after 48 h of algal cultivation (A + R).

Of all the chemicals reported in Table 1, Compound **5** had the highest confidence score of 92.56% for its tentative identification by the NIST14 database as 3-methylhexane, and this identification was confirmed using a pure standard. This simple alkane was highly correlated with only A + R samples throughout the four experiments (Figure 6). For Experiments 1 and 2, the Compound **5** signal was highest during the first 24 h after rotifer addition and then quickly dissipated. Similarly for Experiment 4, the “Day 3, AM” time point reported higher levels of Compound **5**, with signal greatly diminished after only six hours by the time the “Day 3, PM” time point was recorded, further supporting the transience of this chemical signal within algal cultures experiencing rotifer grazing. For Experiment 3, the experiment with the slowest rate of rotifer grazing (Figure 1), the signal from Compound **3** increases slightly between days 3 and 4, but again is gone by day 5. These data suggest that Compound **3** could be a good early, although highly transient, indicator for grazing. Additionally, in Supplementary Table S1, Compound **S6** was tentatively identified as 3-methylhexane and Compound **S4** was tentatively identified as a structural isomer, 2-methylhexane. Both Compounds **S4** and **S6** were similarly correlated to only A + R samples, but in fewer biological replicates than Compound **5**. Compound **S4** was confirmed to be 2-methylhexane with pure standard while Compound **S6** is most likely an isomer of 3-methylhexane since its retention time differed from Compound **5**, which was confirmed as 3-methylhexane. As structural isomers, Compounds **5** and **S4** are likely generated by similar biochemical mechanisms when algae are undergoing wounding events, such as seen here in the samples with active rotifer grazing.

Surprisingly, the only reference to 3-methylhexane as a biomarker is as one of 27 volatiles that are highly correlated and discriminatory between cerumen taken from cancer and healthy human subjects [32]. Possibly, the high levels of reactive oxygen species (ROS) in cancerous cells react with polyunsaturated fatty acids (PUFAs) present within the cell via lipid peroxidation to generate volatile, branched alkanes (including 3-methylhexane, 2-methylhexane, and other alkanes), which are also observed in the breath of cancer patients [33]. It is possible that some of the tentatively identified alkanes with lower replicate and experimental robustness (Compounds **S3**, **S4**, and **S5**, for example) were generated through similar biochemical processes as they are also correlated strongly to A + R samples more than A samples (Supplementary Table S1). In this same way, the PUFAs present in the oleaginous *M. salina* cultures could undergo degradation from ROS upon cellular disintegration by rotifer grazing, resulting in the 3-methylhexane and other alkane signals correlated with A + R samples only.

### 2.6. Compound ***6***

**Figure 7:**
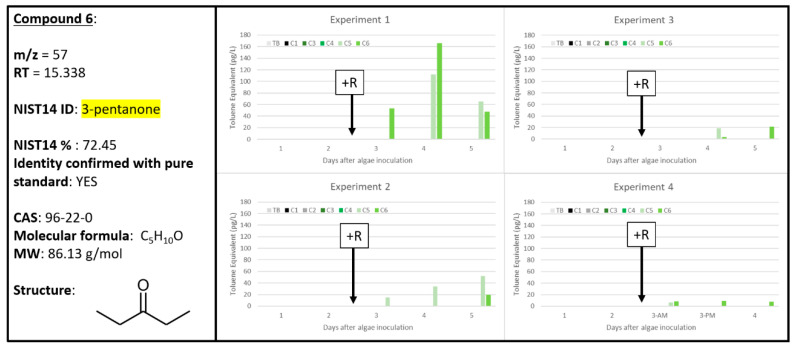
Compound **6** confirmed to be 3-pentanone (96-22-0). Graphs indicate quantity of Compound **6** reported in each of the four experiments in pg L^−1^, where Carboys 1 and 2 (C1, C2) were media blanks (MB) and Carboys 3, 4, 5, and 6 (C3, C4, C5, C6) were inoculated with algae (A) at the beginning of the time course (day 0, data not shown) and only C5 and C6 had rotifers added (+R) after 48 h of algal cultivation (A + R).

The chemical signal most strongly correlated with A + R samples was Compound **6**, which was tentatively identified by NIST14 as 3-pentanone = and subsequently confirmed by analysis of pure compound (Figure 7). Compound **6** was the second chemical signal, besides Compound **5**, to be found robustly (in at least 3 samples) in all four experiments and exclusively in the A + R samples. Additionally, the amount of Compound **6** generated within each experiment scaled with the extent of apparent algal biomass loss to rotifer grazing in each experiment, shown in Figure 1. For example, in Experiment 1, the algal concentration after grazing was the lowest and was correlated with an increase in the concentration of Compound **6** (approximately 160 pg L^−1^) on Day 4. Similarly, the lowest levels of Compound **6** were detected in Experiment 3, which also exhibited the lowest levels of algal biomass loss to grazing. Experiment 3 displayed an increase in Compound **6** over time, and increased duration of the rotifer grazing and algal biomass loss, while Experiment 4 exhibited a relatively flat level of Compound **6**, which was also consistent with the comparably flat rotifer grazing rate observed for that experiment. Regardless of the differences in the rate of algal biomass loss due to rotifers observed between the four experiments (Figure 1), Compound **6** was a reliable signal specifically correlated to samples that were experiencing rotifer grazing. This suggests that Compound **6** could make a useful biomarker indication for algal cultures experiencing grazing.

Interestingly, Compound **S14** was tentatively identified as methyl isobutyl ketone which is a structural isomer of 3-pentanone (Supplementary Table S1). Compound **S14** has a similar correlation with A + R samples in Experiments 1 and 2, but not as strongly for Experiments 3 and 4. Despite the low tentative identification confidence for Compound **6** as 3-pentanone (72.45%, Figure 7), the analytical standard confirmed that Compound **6** is indeed 3-pentanone. There is precedence for 3-pentanone as a byproduct of oxidative stress from plant and algal systems. In *Arabidopsis*, abiotic or biotic stresses often result in oxidative stress and ROS generation, which can be damaging to cellular proteins and biomolecules, including PUFAs. Lipid peroxidation of these PUFAs results in highly reactive unsaturated ketones and aldehydes, which can inhibit or damage biomolecules and/or cellular processes. As a mitigation strategy, the cell utilizes various enzymes to reduce these active chemicals to considerably less toxic and less reactive chemicals: saturated ketones, including 3-pentanone [34]. Other known biological sources of 3-pentanone include the smooth Cayenne pineapple [35] and endophytic fungi [36]. In addition to the 2-methylfuran detection from Amaranth residues [27], 3-pentanone was emitted from all amaranths tested and found to have a potent allelochemical effect of >90% germination inhibition for tomato seedlings. Additionally, 3-pentanone was found as a volatile produced by algal blooms of the Chrysophyte, *Synura petersenii* [37]. Thus, it is plausible that *Microchloropsis salina* produced 3-pentanone in the presence of active rotifer grazing as an infochemical for other algal cells. Further studies are required to confirm the biological effect of 3-pentanone on neighboring algal cells undergoing algal grazing.

### 2.7. Compound ***7***

**Figure 8:**
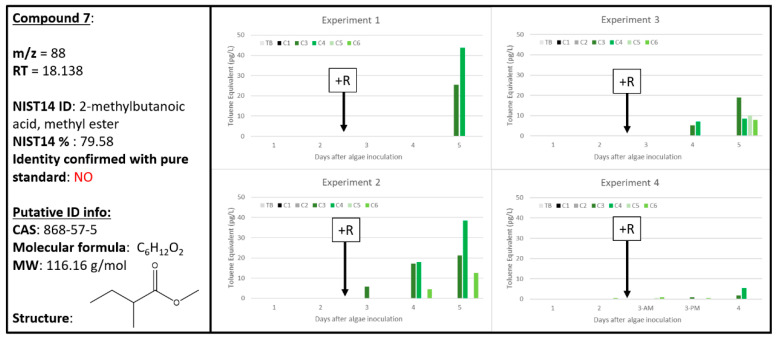
Compound **7** tentatively identified by NIST14 to be the methyl ester of 2-methylbutanoic acid (868-57.5). Graphs indicate quantity of Compound **7** reported in each of the four experiments in pg L^−1^, where Carboys 1 and 2 (C1, C2) were media blanks (MB) and Carboys 3, 4, 5, and 6 (C3, C4, C5, C6) were inoculated with algae (A) at the beginning of the time course (day 0, data not shown) and only C5 and C6 had rotifers added (+R) after 48 h of algal cultivation (A + R).

Predominantly found in algae only (A) samples, Compound **7** was tentatively identified as the methyl ester form of 2-methylbutanoic acid (Figure 8). All four experiments observed higher signal for Compound **7** as correlated with higher algal concentrations over time, especially for Experiments 1 and 2. Experiments 2 and 3 also indicated the presence of Compound **7** within A + R samples for the later time points. This could be because these A + R samples still had actively growing, healthy algal cells among the algae experiencing rotifer grazing. This is especially true for Experiment 3, which exhibited the lowest rate of biomass loss due to rotifer grazing (Figure 1). Experiment 4 also indicated an increase in Compound **7** for A only samples, but A + R samples on Day 2 and 3 had very low levels of Compound **7** present. To a much lesser extent, some A + R samples also increased in abundance of Compound **7** over time, such as for Experiments 2 and 3. This complicates the use of Compound **7** as a clear bioindicator of healthy algal cells. Although, if there are some healthy cells present in a culture (such as those with low apparent grazing rates, such as in Experiment 3), then the presence of Compound **7** is expected. For use as a biomarker for algal health or stress/death, Compound **7** alone might not be the best signal to track the presence of grazer-induced stress.

The tentative identification of Compound **7** is the methyl ester of the organic acid 2-methylbutanoic acid (Figure 8), which is structurally related to the methyl butyrate (chemically different by only one methyl group), a known fruit aroma chemical [35]. Plants are known to emit a variety of esters that have found their way into various perfumes and fragrances due to their fruity, sweet aroma. Organic acids can be converted to its related ester through the addition of an alcohol [38]. Many plant-based esters are found in essential oils and are generally associated with various therapeutic properties, including antifungal, anti-inflammatory, and antispasmodic. For Senga Sengana strawberries, the organic acid, 2-methyl butanoic acid, was found to be part of the aromatic profile [39]. For these reasons, it is plausible that the identity of Compound **7** is the methyl ester of 2-methylbutanoic acid. This publication would be the first tentative identification of the methyl ester of 2-methylbutanoic acid in algal cultures.

### 2.8. Ambiguous Chemical Signals, Compounds ***8***, ***9***, ***10***

**Figure 9:**
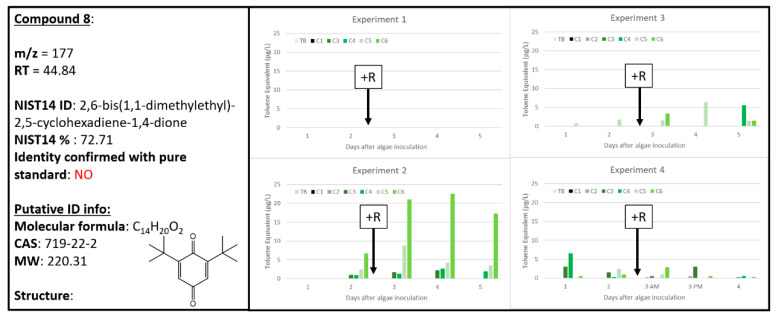
Compound **8** tentatively identified by NIST14 to be 2,6-bis(1,1-dimethylethyl)-2,5-cyclohexadiene-1,4-dione (CAS: 719-22-2). Graphs indicate quantity of Compound **8** reported in each of the four experiments in pg L^−1^, where Carboys 1 and 2 (C1, C2) were media blanks (MB) and Carboys 3, 4, 5, and 6 (C3, C4, C5, C6) were inoculated with algae (A) at the beginning of the time course (day 0, data not shown) and only C5 and C6 had rotifers added (+R) after 48 h of algal cultivation (A + R).

Despite passing the conservative thresholds for detection within the four experiments, Compounds **8**, **9**, and **10** all have ambiguous abundance and distribution patterns that make it difficult to utilize these chemicals as biomarkers of algal health or stress/death. Compound **8**, tentatively identified as 2,6-bis(1,1-dimethylethyl)-2,5-cyclohexadiene-1,4-dione, was absent from all Experiment 1 samples, highly abundant in one A + R sample in Experiment 2, and was detected at lower levels in A and A + R samples in Experiments 2–4, although with no apparent pattern (Figure 9). Also known as 2,6-di-tert-butyl-P-benzoquinone, Compound **8**′s tentative chemical identification could be explained as a byproduct of cellular oxidative stress—quinones are commonly used by biological organisms to reduce ROS intracellularly. However, the lack of consistency with a single biological sample type (A or A + R) does not support the use of Compound **8** as a useful biomarker of algal health.

**Figure 10:**
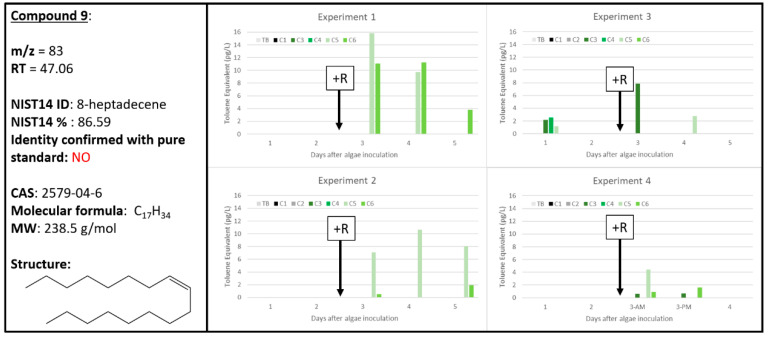
Compound **9** tentatively identified by NIST14 to be 8-heptadecane (CAS: 2579-04-6). Graphs indicate quantity of Compound **9** reported in each of the four experiments in pg L^−1^, where Carboys 1 and 2 (C1, C2) were media blanks (MB) and Carboys 3, 4, 5, and 6 (C3, C4, C5, C6) were inoculated with algae (A) at the beginning of the time course (day 0, data not shown) and only C5 and C6 had rotifers added (+R) after 48 h of algal cultivation (A + R).

With a relatively high confidence of 86.59%, the NIST14 database tentatively identified Compound **9** as 8-heptadecene, which does have precedence in the literature as a known biogenic VOC (Figure 10). Not only has 8-heptadecene been found as an algal volatile from two brown algae, *Padina pavonia* (L.) Gaill and *Hydroclathrus clathrus* (C. Agardh) Howe, the volatile mixtures from these algae were also found to have antimicrobial activity (to varying degrees) against a panel of microbes [40]. Additionally, 8-heptadecene was found to be the major volatile produced by the marine green alga, *Bryopsis maxima*, after mechanical wounding [41] and during various growth phases by *Nannochloropsis* and other algae [15]. Unfortunately, our data for Compound **9** do not show a similarly strong correlation with rotifer grazing. Experiments 1 and 2 exclusively observe Compound **9**, tentatively 8-heptadecene, with A + R samples. However, Experiments 3 and 4 indicated both A and A + R samples generated 8-heptadecene, in variable quantities with no distinct pattern. Additional evidence is required to characterize Compound **9** as a biomarker for wounding of *Microchloropsis salina* algal cultures.

**Figure 11:**
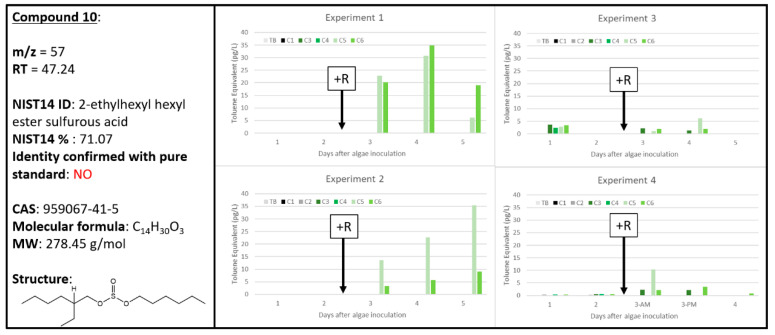
Compound **10** tentatively identified by NIST14 to be 2-ethylhexyl hexyl ester sulfurous acid (CAS: 1000309-20-2). Graphs indicate quantity of Compound **10** reported in each of the four experiments in pg L^−1^, where Carboys 1 and 2 (C1, C2) were media blanks (MB) and Carboys 3, 4, 5, and 6 (C3, C4, C5, C6) were inoculated with algae (A) at the beginning of the time course (day 0, data not shown) and only C5 and C6 had rotifers added (+R) after 48 h of algal cultivation (A + R).

Compound **10** was tentatively identified as 2-ethylhexyl hexyl ester sulfurous acid albeit with a relatively low NIST14 confidence score of 71.07% (Figure 11). Interestingly, as seen with Compound **9**, Compound **10** was highly correlated to A + R samples in Experiments 1 and 2, but this relationship was not replicated in Experiments 3 and 4. Potentially, there were unknown biological conditions in Experiment 1 and 2 that resulted in Compounds **9** and **10** being highly correlated to A + R samples, but not in Experiments 3 and 4. The only tenuous link to plant-based volatile literature for Compound **10**′s tentative identification as 2-ethylhexyl hexyl ester sulfurous acid is its detection in the vinegar-processed medicinal herb, *Curcuma zedoaria* [42]. Like Compounds **8** and **9**, additional wounding experiments of *Microchloropsis salina* need to confirm whether Compound **10** is useful as a biomarker of algal health. 

### 2.9. Less Robust Compounds ***S1–S50*** of Note

There were 50 chemicals that were not defined as “robustly” present within at least three samples in at least three of the four experiments (Supplementary Table S1). Despite not meeting this threshold, there were some chemicals that were very close and others that have been previously correlated with algal wounding that are worth mentioning. Compounds **S28** and **S45** were tentatively identified as 2,2,6-trimethyl-cyclohexanone (NIST14 confidence score 71.2%) and 4-(2,6,6-trimethyl-1-cyclo-hexen-1-yl)-2-butanone (more commonly known as dihydro-β-ionone with a NIST14 confidence score of 76.4%), respectively, and both were only associated with A + R samples. Additionally, trans-β-ionone was confirmed with pure analytical standard to be compound **S46** and is also identified in this work as selectively correlated with A + R samples. Compounds **S28**, **S45**, and **S46** were previously identified biomarkers of *M. salina* grazing [17] albeit much more robustly than in this current work. The differences in chemical profiling performed herein (using Carbopack B/X™ sorption tubes and active sampling) and Reese et al. [17] (solid phase microextraction fibers and passive sampling) can account for this difference in chemical detection. These differences justify the use of orthogonal sampling techniques in order to understand the broader chemical landscape for a biological system.

Several other known algal-generated volatiles were detected, although not at the threshold determined to be suitable as a biomarker (Supplementary Table S1). Compound **S15**, tentatively identified as 3-hexanone (NIST14 confidence score 82.4%) has been found in volatile samples collected from the brown alga, *Ectocarpus siliculosus*, previously [43]. Our data show Compound **S15** as strongly correlated to the A + R samples, but only for in Experiments 1 and 2. Compound **S16** was confirmed to be octane and was strongly correlated with five of the six A + R samples for Experiment 1. Octane has been previously identified from Amaranth residue [27] as well as from algae [28]. Compound **S21** was tentatively identified to be 2-acetyl-5-methylfuran (NIST14 confidence score 72.4%), which is a known volatile found in many foods and plants, such as potato, coffee, tomato, and cereals [44]. It is speculated that 2-acetyl-5-methylfuran would make a good biomarker for consumption of these foods [44], and similarly, Compound S21 is only found in A + R samples where algae are being actively consumed. 

Compound **S12** was tentatively identified to be dimethyl sulfide (DMS) through AMDIS deconvolution and searched against the NIST14 database. There exists a considerable number of published works characterizing DMS as an important algal metabolite, allelochemical, and chemoattractant known for its substantial impact on the global biogeochemistry of sulfur (for review, see [45]). Many other chemicals that were not able to be identified by the NIST14 database at ≥70% confidence score were still clearly associated with A + R samples, such as Compounds **S19**, **S32**, and **S47**. Potentially, these compounds would benefit from sampling via an alternative sorptive material(s) to facilitate better detection and identification.

## 3. Materials and Methods

### 3.1. Microalgae Culture, Rotifer Culture, and Experimental Setup

The three experiments described in [17] were also utilized for this work (Experiments 1–3) including the daily cell measurements collected using chlorophyll fluorescence (Figure 1). Duplicate fluorescence measurements for each sample were collected for each time point and averaged, as in [17], and then normalized to the highest final fluorescence for the *M. salina* control in the absence of rotifers. In addition to the three experiments detailed in [17], a fourth experiment (Experiment 4) was conducted for this present work with the same methodology, except that volatile samples were collected twice on the same day during the 24 h period immediately following rotifer addition (see Figure 1 for headspace sampling time points indicated by purple dot; •).

### 3.2. Active Sampling Procedure of Algal Culture Headspace

In addition to the passive sampling of algal culture headspace performed in [17], active headspace sampling was performed on the same experiments. VOCs were collected onto multi-bed sorbent tubes (Supelco, Bellefonte, USA) with primary bed of Carbopack B sorbent backed with a section of Carbopack X. Prior to use, the sorbent tubes were conditioned at 345 °C for 30 min with a helium purge (30 cc min^−1^) then sealed in Teflon capped tubes at Lawrence Berkeley National Laboratory (LBNL). Samples were shipped on ice packs to Sandia National Laboratories (SNL) for sample collection. The headspace over culture was continuously purged at 100 cc min^−1^ using sparging gas 1% CO_2_ in air (20% oxygen in nitrogen). Algal headspace samples were collected on these Carbopack B/X™ sorption tubes using an oil-free vacuum pump pulling at 90 cc min^−1^ (equivalent to 90 mL min^−1^). Carbopack B/X™ sorption tube sampling was collected simultaneously from each of the six carboys shown in the experimental set up in [17], where carboys 1 and 2 (C1, C2) were media blanks, carboys 3 and 4 (C3, C4) contained algae only, and carboys 5 and 6 (C5, C6) were algae cultures that had 88 rotifers mL^−1^ added after 48 h of cultivation. Additionally, one travel blank was exposed briefly via passive sampling to the room air, cooler, and freezer that the rest of the samples experienced. To determine optimal sampling time for metabolomics analysis, different sampling times were used for the four experiments: 30 min for Experiment 1, 60 min for Experiments 2 and 3, and 120 min for Experiment 4. Exposed Carbopack B/X™ sorption tubes were sealed with Teflon lined caps and stored on ice packs until shipment to LBNL. Upon return to LBNL, samples were stored in a freezer until analysis (within 2 weeks of arrival). 

### 3.3. GCMS Data Acquisition

Before analysis, each sample was purged at room temperature with a flow of dry He for 10 min at a flow of 30 cc min^−1^. A gas-phase internal standard (120 ng of 1-bromo-4-fluorobenzene, Sigma) was injected into each sorbent tube followed by an additional 4 min purge with helium (30 cc min^−1^) at room temperature. Once prepared, the sorbent tubes were analyzed by gas chromatography–mass spectrometry (GC-MS) using the following thermal desorption injection system: a ThermoDesorption Autosampler (Model TDSA2; Gerstel, Linthicum, USA), a thermal desorption oven (Model TDS3, Gerstel) and a cryogenically cooled injection system (Model CIS4; Gerstel). The cooled injection system contained a Tenax-TA©-packed glass injection liner (P/N 013247- 005-00; Gerstel). The samples were desorbed at 50 cc/min (splitless) using the following temperature profile: 25 °C (0.5 min delay) followed by a 60 °C min^−1^ ramp to 330 °C with a 1 min hold time. The cooled inlet was held at 1 °C to focus the sample and then heated after 0.1 min to 300 °C at a rate of 12 °C s^−1^, followed by a 2 min hold time to inject the sample. The GC was operated in the solvent vent mode with a splitless injection. Compounds were resolved on a GC (Series 6890 Plus; Agilent Technologies) equipped with a 30 m in height by 0.25 mm in diameter Restek Rxi-624Sil MS capillary column with a 0.14 mm film thickness (cat# 13868). The initial oven temperature was 1 °C, held for 2 min, then increased to 100 °C at 5 °C min^−1^ (hold 2 min), increased to 140 °C at 3 °C min^−1^, then increased to 300 °C at 10 °C min^−1^ and held for 10 min. The helium flow through the column was held constant at 1.2 mL min^−1^ (initial pressure 47 kPa, 39 cm s^−1^). The resolved analytes were detected using electron impact MS (5973; Agilent Technologies, Santa Clara, USA) operated in total ion current (TIC) mode with target and qualifier ions specified for each target compound. The MS temperature settings were 240, 230, and 150 °C for the transfer line, MS source, and MS quad, respectively. The MS was operated in scan mode with a range of 34 *m*/*z* to 450 *m*/*z*.

### 3.4. Metabolomics Analysis of GCMS Data

Data collected from Carbopack B/X™ sorption tubes and analyzed via GCMS were then processed similarly to previous work [17], with some select software and parameter differences listed herein. Original ChemStation data files were translated using MassHunter GC/MS Translator B.07.03 2127 (15 May 2015) before processing for chromatographic deconvolution using Agilent MassHunter Qualitative Analysis Workflows B.08.00 with the same parameters as used in [17]: a retention time window size factor of 90.0, a signal-to-noise threshold of 2.00, an absolute ion height filter of 1000 counts, and ≥5 ions required for compound detection. Exported CEF files were transported to Mass Profiler Professional (MPP) version 15.1 for alignment across all samples from all four experiments (108 samples total; 26 for Experiment 1; 25 for Experiment 2, 30 for Experiment 3; 27 for Experiment 4) using a retention time tolerance of 0.15 min, a mass spectral match factor of 0.6/1.0, a delta *m*/*z* tolerance of 0.2 Da, and with a Pareto statistical baseline correction. From this processing, 1266 compounds were aligned for tentative identification using National Institute of Standards and Technology 2014 (NIST14) database with a minimum requirement for ≥70% spectral match using ≥10 ion counts for peak filtering through the NIST14 library. Compound identification in this way was based initially on mass spectra present in the NIST14 database. Biomarkers of importance to Algae and/or Algae + Rotifer cultures were selected from the list of 1266 chemicals by filtering to remove chemicals that were only present in no more than 2 samples of the 108 experimental samples and/or were only present in blanks (834 chemicals removed). For the remaining 432 chemicals, the signals were scaled according to sampling time differences across experiments: Experiment 1, 30 min; Experiment 2 and 3, 60 min; Experiment 4, 120 min. To normalize signal intensities across all four experiments, an external mathematical scalar of 0.5 was applied to all Experiment 2 and 3 signal intensities and a scalar of 0.25 was applied to Experiment 4 signal intensities. Based on these normalized signal intensities, calculations were performed to determine Algae (A) or Algae + Rotifer (A + R) signals that were at least 10-fold higher than signals generated by the respective blanks in the experiment. This process facilitated down selection to 178 compounds and, of these, only 60 compounds were present in more than one of the four experiments. These 60 compounds were separated by reproducibility within an experiment—compounds identified at least three times within a single experiment *and* found in three of the four experiments were distinguished as “robust” chemical signals and summarized in Table 1, while the remainder were labeled as “less robust” and are summarized in Supplementary Table S1.

The 60 compounds initially identified using the metabolomics software and subsequently filtered down from the initial 1266 compounds were further evaluated as follows. Specific samples (GCMS data files) were selected based on the presence of the specific base peak *m*/*z* at the RT and the overlay function in ChemStation Enviroquant was used to identify the sample, or subset of samples, with the highest abundance. If the peak in the selected sample was significantly greater than background and well shaped, then the spectrum was searched in the NIST14 database directly. If the best peak for a given *m*/*z* RT combination was low and/or clearly consisted of coeluting compounds, then the spectrum was imported into the Automated Mass Spectral Deconvolution and Identification System (AMDIS) software and deconvoluted. The spectrum associated with the identified base peak *m*/*z* for the compound was searched against the NIST14 database to provide improved identification, if possible. For a subset of the identified compounds, focused mainly on the robust set, pure standards were run on the instrument and the resulting mass spectra and retention time were used to confirm or refute the tentatively identified compounds. 

Several authentic chemical standards were purchased (Sigma-Aldrich, St. Louis, MO, USA) to confirm/refute tentative identifications. These included pentane (99%), 1,4-pentadiene (99%), isoprene (99%), *trans*-1,3-pentadiene (90%), 2-methylfuran (99%), 3-methylfuran (100%), 2-methylhexane (99%), 3-methylhexane (99%), 3-pentanone (99%), octane (99%), and beta-ionone (99%). Pentane, octane and beta-ionone were diluted in methanol (99%) (Sigma-Aldrich, St. Louis, MO, USA) and injected on thermal desorption tubes then purged for 5 min at 30 mL/min (to remove methanol) prior to analysis. The remaining standards were injected (2 L) individually into a 2 L standard dilution bulb at room temperature then 200 L of dilute gas was injected onto a thermal desorption tube and purged 3 min at 30 mL/min (to load standard onto tube) prior to analysis.

### 3.5. Quantitative Analysis of Chemical Data

All Carbopack B/X™ sorption tubes were spiked with 120 ng of p-bromofluorobenzene (CAS: 460-00-4) just prior to analysis as indicated above as an internal standard to normalize detector response. Semi-quantitative data were obtained from a 5-point calibration curve of pure standard (toluene, CAS: 108-88-3; Sigma, St. Louis, MO, USA) following EPA methods [20]. The toluene equivalent was calculated using this standard for the 10 “robust” chemical signals (Table 1) by using the calibration equation:

$$Y=\left(7\times 10^{-16}\right)X^{2}+\left(4\times 10^{-7}\right)X$$

where “*X*” represents the “response” for each of the chemical signals and “*Y*” represents ng of toluene and the regression is based on the total ion current calibration of toluene specific to the instrument used in this study. To determine the concentration (in pg L^−1^) of each chemical in the dynamic head space, or response per volume (L) of air that has sparged through the culture, these values were then divided by the product of the controlled sample flow rate (0.1 L min^−1^) and the sample duration (30, 60 or 120 min). These calculations and the respective graphs (Figure 2, Figure 3, Figure 4, Figure 5, Figure 6, Figure 7, Figure 8, Figure 9, Figure 10 and Figure 11) were generated using Microsoft Excel 2016 software.

## 4. Conclusions

With the increasing energy needs of our nation, it is imperative to develop robust and sustainable, renewable energy sources, such as biofuel, which are often produced from complex biological systems. Using volatiles as an indicator, and eventually a predictor, of the health or state of a biological system is still in its infancy. Our work supports and expands upon previous work that specific chemicals are generated only in the presence of active rotifer grazing. Our results show that Compounds **1**, **3**, **5**, and **6** are most robustly correlative with A+R samples (Table 1). These chemicals were identified as pentane, 2-methylfuran, 3-methylhexane, and 3-pentanone, respectively, and are described for the first time as signals generated by *M. salina* cultures in the presence of the grazer, *B. plicatilis.* These algal metabolites, in combination with the carotenoid breakdown products previously identified from these experiments [17], can be used collectively to aid in the diagnostic evaluation of algal mass cultures. With the use of computational modeling and machine learning, future work to create predicative models based on volatile detection can be pursued to aid in the regulation of complex biological systems. Additionally, advanced technology to specifically identify biomarkers of algal stress/death can be developed for use by the algal industry. In this way, our work contributes to a growing chemical library to best describe, diagnose, and cultivate algae mass culture systems.

## Figures and Tables

**Table 1:** Robust VOCs present in replicate experiments. Compounds **1**–**10** represent VOCs that are found to be 10-fold higher in algae (A) and/or algae with rotifer (A + R) samples than in the blanks (B) and robustly found in at least three or more samples in at least three of the four experiments. Number of blank (B), A, and A + R samples that were found to contain each of the compounds are enumerated for each Experiment, with the total sample size (n) for each sample type listed at the bottom of each column. The darkness of the green reflects the number of samples where each chemical was found. The mass-to-charge (*m*/*z*) for the base peak ion and retention time (RT, in minutes) for Compounds **1**–**10** are shown. Tentative compound identifications (ID) by the NIST14 database with a confidence of ≥70% (NIST14%) are shown along with the CAS number for each. Confirmed compound identification is denoted with yellow highlight and unconfirmed compounds are shown with red font.

	Experiment 1			Experiment 2			Experiment 3			Experiment 4								
Sampling Time:	30 min			60 min			60 min			120 min					Tentative Compound ID, CAS, and NIST14 %			Confirmed ID CAS
Compound #	B	A	AR	B	A	AR	B	A	AR	B	A	AR	m/z	RT	Compound ID	CAS	NIST14 %	
1	0	0	3	0	0	5	0	0	5	0	0	1	43	5.730	pentane	109-66-0	90.45	**Pentane** 109-66-0
2	0	13	2	0	10	5	1	13	6	0	8	1	67	6.330	1,4-pentadiene	591-93-5	84.80	**2-methyl-1,3-butadiene** 78-79-5
3	0	0	5	1	0	6	0	0	6	0	0	1	82	10.409	2-methylfuran	534-22-5	79.74	**2-methylfuran** 534-22-5
4	0	6	5	0	10	6	1	10	6	0	10	4	82	10.920	2-methylfuran	534-22-5	79.56	**3-methylfuran** 930-27-8
5	0	0	4	0	0	3	0	0	4	0	0	3	71	12.930	3-methylhexane	589-34-4	92.56	**3-methylhexane** 589-34-4
6	0	0	5	0	0	4	0	0	3	0	0	4	57	15.338	3-pentanone	96-22-0	72.45	**3-pentanone** 96-22-0
7	0	2	0	0	5	2	0	4	2	0	4	3	88	18.138	2-methylbutanoic acid methyl ester	868-57-5	79.58	**unconfirmed**
8	0	0	0	0	9	6	0	3	5	2	11	4	177	44.841	2,6-bis(1,1-dimethylethyl)-2,5-cyclohexadiene-1,4-dione	719-22-2	72.71	**unconfirmed**
9	0	0	5	0	0	5	0	4	1	0	2	3	83	47.058	8-heptadecene	2579-04-6	86.59	**unconfirmed**
10	0	0	6	0	0	6	0	6	4	2	8	4	57	47.239	2-ethylhexyl hexyl ester sulfurous acid	959067-41-5	71.07	**unconfirmed**
sample size (n)	6	14	6	9	10	6	11	13	6	10	12	5						

## Supplementary Materials

The following are available online at https://www.mdpi.com/2218-1989/10/9/361/s1: Supplemental Table S1: List of 50 less “robust” VOCs; Supplemental Figure S1: Extracted chromatograms for Compounds 2 and S2; Supplemental Figure S2: NIST14 references for 1,4-pentadiene (A) and isoprene (B); Supplemental Figure S3: Comparison of extracted chromatograms for Compound 2 (*m*/*z* 67, RT 6.330) and Compound S2 (*m*/*z* 67, RT 7.525) to the NIST14 reference for 1,4-pentadiene (A) and NIST14 reference for isoprene (B); Supplemental Figure S4: NIST14 reference for 2-methylfuran (A) with the extracted chromatograms at 82 *m*/*z* at RT 10.409 for Compound 3 (B) and 82 *m*/*z* at RT 10.920 for Compound 4 (C); Supplemental Figure S5: Comparison of extracted chromatograms for Compound 3 at *m*/*z* 82 at RT 10.920 and Compound 4 at *m*/*z* 82 at RT 10.409 to the NIST14 reference for 2-methylfuran.

## Copyright Notice

This manuscript has been authored by an author at Lawrence Berkeley National Laboratory under Contract No. DE-AC02-05CH11231 with the U.S. Department of Energy. The U.S. Government retains, and the publisher, by accepting the article for publication, acknowledges, that the U.S. Government retains a non-exclusive, paid-up, irrevocable, world-wide license to publish or reproduce the published form of this manuscript, or allow others to do so, for U.S. Government purposes.

## Abbreviations

The following abbreviations are used in this manuscript:

A	algae cultures (Microchloropsis salina)
A+R	algae cultures (*Microchloropsis salina*) in the presence of marine rotifers (*Brachionus plicatilis*)
AMDIS	Automated Mass Spectral Deconvolution and Identification System
C1, C2	Carboy 1 and Carboy 2, containing medium only
C3, C4	Carboy 3 and Carboy 4, containing algae only
C5, C6	Carboy 5 and Carboy 6, containing algae cultures that had rotifers added after 48 h of cultivation
CEF	compound exchange format
GCMS	gas chromatography–mass spectrometry
MPP	Mass Profiler Professional
NIST14	National Institutes of Standards and Technology 2014
PUFAs	polyunsaturated fatty acids
ROS	reactive oxygen species
RT	retention time
VOC	volatile organic compound
