# Supplementary material for: Low Molecular Weight Volatile Organic Compounds Indicate Grazing by the Marine Rotifer Brachionus plicatilis on the Microalgae Microchloropsis salina
Source: Metabolites. 2020 Sep 4;10(9):361. doi: 10.3390/metabo10090361 (PMC7570158; doi:10.3390/metabo10090361)
Supplement: Supplementary file 1 [file metabolites-10-00361-s001.pdf]

Supplemental Table 1: **List of 50 less "robust" VOCs.** Compounds S1-S50 represent VOCs that are found to be ten-times higher in algae (A) and/or algae with rotifer (A+R) samples than in the blanks (B), but are not as "robust" since they are found in fewer than three experiments or fewer than three replicates per experiment. Number of blank (B), A, and A+R samples that were found to contain each of the compounds are enumerated for each Experiment, with the total sample size (n) for each sample type listed at the bottom of each column.The base peak mass-to-charge (m/z) and retention time (RT, in minutes) for Compounds S1-S50 are shown. Shown here are tentative compound identifications (ID) made by Agilent's MPP deconvolution software and searched against the NIST14 database with a confidence of ≥70% . Additionally, shown here are tentative compound ID made by AMDIS deconvolution and searched against NIST14 with probability of ≥70% or the results from the analysis of pure standards. Confirmed compound identification by pure standards is denoted with yellow highlight, consistent chemical identification by MPP and AMDIS are shown with light blue highlight, and refuted compound identifications (by comparison to pure standards) are shown with red font. Blank cells represent low confidence (under 70%) tentative compound identifications by MPP or AMDIS and were not confirmed by any tested pure standards.

sampling time:	Experiment 1 30 min			Experiment 2 60 min			Experiment 3 60 min			Experiment 4 120 min						
**compound #**	**B**	**A**	**AR**	**B**	**A**	**AR**	**B**	**A**	**AR**	**B**	**A**	**AR**	**m/z**	**RT**	**MPP deconvolution, tentative compound ID against NIST14 (% confidence)**	**AMDIS deconvolution, tentative compound ID against NIST14 (% probability)**
S1	0	0	0	0	1	2	0	1	0	0	0	0	72	6.357		
S2	0	0	0	0	0	3	0	0	0	0	0	1	67	7.525	1,4-pentadiene (78.4)	refuted with pure standard; likely an isomer
S3	0	0	3	0	0	5	0	0	0	0	0	1	69	10.088	2,3-dimethyl-1-butene (72.2)	
S4	0	0	3	0	0	2	0	0	3	0	0	1	85	12.522	2-methylhexane (84.5)	confirmed as: **2-methylhexane**
S5	0	0	2	0	0	3	0	0	1	0	0	1	56	12.649	2,2,3,4-tetramethylpentane (88.9)	2,3-dimethylpentane or isomer (70)
S6	0	0	1	0	0	1	0	0	1	0	0	3	70	13.337	3-methylhexane (86.5)	refuted with pure standard; likely an isomer
S7	0	0	1	0	0	5	0	0	1	0	0	1	81	14.573	dimethylphosphinic fluoride (74.3)	2-ethylfuran (75)
S8	0	0	4	0	0	2	0	0	3	0	2	1	55	14.900	(R)-(+)-3-methylcyclopentanone (76.4)	1-pentene-3-one (85)
S9	0	2	0	2	4	4	1	1	0	0	1	0	96	15.222	2,4-dimethylfuran (73.7)	2,4-dimethylfuran (85)
S10	0	0	1	0	0	2	0	0	1	0	0	1	85	15.387	2-pentoxy-tetrahydropyran (82.0)	
S11	1	2	2	3	5	6	0	0	0	0	0	1	57	15.831	2-methyl-3-pentanone (75.1)	2,2-dimethyl-propanoic acid methyl ester (84)
S12	0	0	0	2	4	4	0	0	3	0	5	0	94	16.831		dimethylsulfide (97.6)
S13	1	0	1	0	1	0	0	0	1	0	0	1	79	17.183		
S14	0	2	4	2	2	4	0	0	1	0	0	0	43	17.189	methyl isobutyl ketone (87.5)	
S15	0	0	4	0	0	3	0	0	0	0	0	1	57	17.512	3-hexanone (82.4)	2-methyl-3-pentanone (70)
S16	0	0	5	0	1	1	0	0	0	0	0	0	85	18.177	octane (81.2)	confirmed as: **octane**
S17	0	0	2	0	0	2	0	0	0	0	0	0	69	18.201	1-cyclopropyl-1-propanone (80.8)	
S18	0	0	0	0	1	0	3	4	3	0	2	3	129	19.498	bromochloronitromethane (75.4)	bromodibromochloromethane (91)
S19	0	0	0	0	0	4	0	0	2	0	0	2	107	20.231		
S20	0	0	5	0	0	3	0	0	0	0	0	2	109	20.748		
S21	0	0	3	0	0	2	0	0	0	0	0	4	109	20.967	2-acetyl-5-methylfuran (72.4)	
S22	0	0	0	0	1	0	4	3	4	0	2	3	173	23.222	tribromo-methane (82.6)	tribromo-methane (92)
S23	0	1	2	0	1	0	0	0	0	0	0	0	97	27.348		2,2,4,6,6-pentamethyl-3-heptene (84)
S24	0	0	1	0	1	0	0	0	3	2	0	3	57	27.762		3-octanone (78)
S25	0	0	0	0	0	0	0	0	1	0	0	2	108	27.900		6-methyl-5-heptene-2-one (83)
S26	0	0	0	1	0	0	0	2	1	0	0	1	55	28.704		
S27	0	0	0	0	0	1	5	8	5	0	6	4	102	29.786	2-ethylhexanoic acid, ethyl ester (73.6)	2-ethylhexanoic acid, ethyl ester (93)
S28	0	0	1	0	0	2	0	0	0	0	0	0	82	30.527	2,2,6-trimethyl-cyclohexanone (71.2)	2,2,6-trimethyl-cyclohexanone (89)
S29	0	0	1	0	0	1	0	0	0	0	0	2	69	30.715		
S30	0	0	0	0	0	1	0	0	1	0	0	1	82	31.719		
S31	0	0	0	0	0	3	0	0	1	0	0	3	133	32.747	1-(2,3-dimethylphenyl)ethanone (74.0)	
S32	0	0	0	0	0	4	0	0	0	0	0	4	135	33.265		
S33	0	0	0	0	0	1	0	4	2	0	1	0	96	35.041		
S34	0	0	0	0	2	0	0	1	1	9	12	4	71	39.550	dodecyl 2-ethylhexyl ester sulfurous acid (75.0)	
S35	0	0	0	0	3	1	0	1	1	2	2	0	71	41.307		2-ethylhexyl ester n-butyric acid (88)
S36	0	1	1	0	0	2	0	2	2	5	10	2	142	41.529		
S37	0	1	0	0	0	1	0	0	0	1	8	2	71	42.138		
S38	0	0	0	0	0	3	0	0	1	0	0	2	175	42.346	4-(1,1-dimethylethyl)-benzenepropanal (73.2)	
S39	0	0	0	0	0	3	0	0	0	0	0	1	159	42.438		
S40	0	0	0	0	0	3	0	0	2	0	0	4	157	42.488	1,2-dihydro-4,5,7-trimethyl-naphthalene (72.0)	
S41	0	2	0	0	0	2	0	0	0	0	0	0	138	42.755		
S42	0	0	1	0	0	0	0	0	0	0	2	1	154	43.237		
S43	1	0	0	1	0	0	0	1	2	0	7	1	170	43.550		
S44	0	2	2	0	0	2	0	0	1	3	9	3	71	43.763		
S45	0	0	1	0	0	4	0	0	0	0	0	2	121	44.385	4-(2,6,6-trimethyl-1-cyclohexen-1-yl)-2-butanone (76.4)	4-(2,6,6-trimethyl-1-cyclohexen-1-yl)-2-butanone (77)
S46	0	0	1	0	0	4	0	0	3	0	0	1	177	45.243		confirmed as: **beta-ionone**
S47	0	0	0	0	0	2	0	0	3	0	0	0	173	47.232		
S48	0	0	0	0	1	0	2	0	0	0	4	0	71	49.857	3-ethyl-3-methyl-decane (72.6)	
S49	0	0	0	0	1	0	2	0	0	0	4	0	71	50.290	1-iodo-dodecane (72.8)	
S50	0	5	4	0	1	1	2	6	1	4	0	0	55	56.776	oleanitrile (70.7)	oleanitrile (65)
sample size (n)	6	14	6	9	10	6	11	13	6	10	12	5				

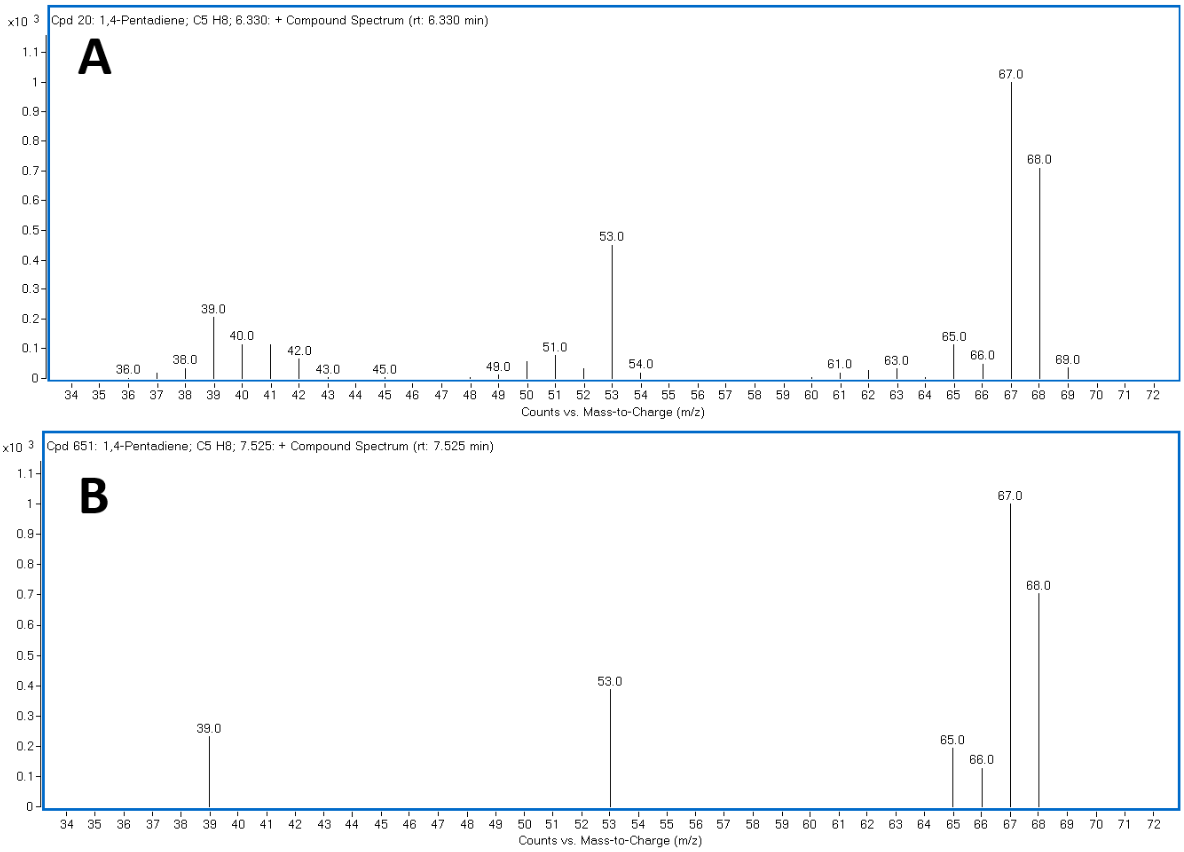

Supplemental Figure 1: **Extracted chromatograms for Compounds 2 and S2.** Compound 2 has a base peak at 67 m/z for RT 6.330 min (**A**) and Compound S2 has a base peak at 67 m/z for RT 7.525 min (**B**).

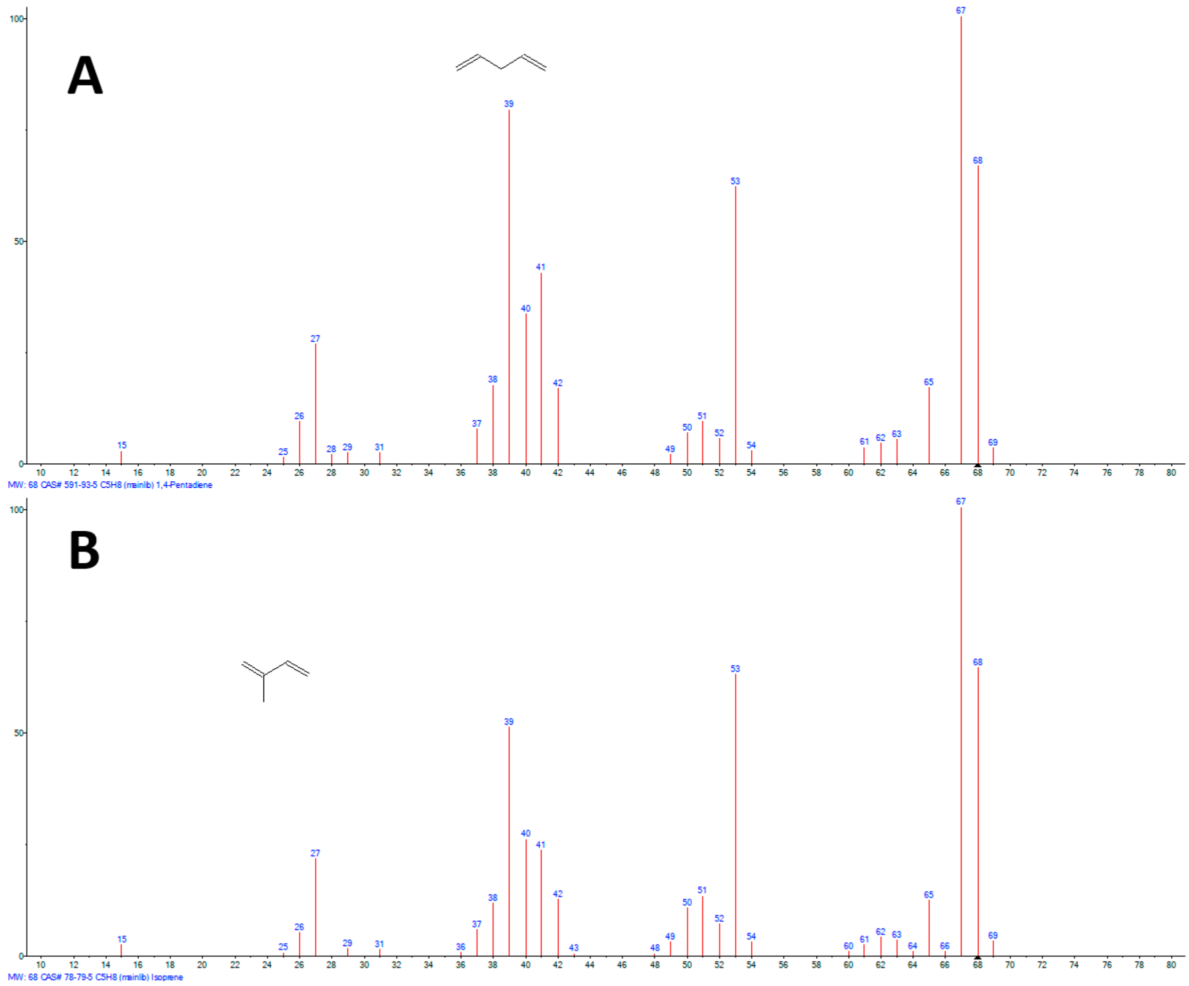

Supplemental Figure 2: NIST14 references for 1,4-pentadiene (**A**) and isoprene (**B**).

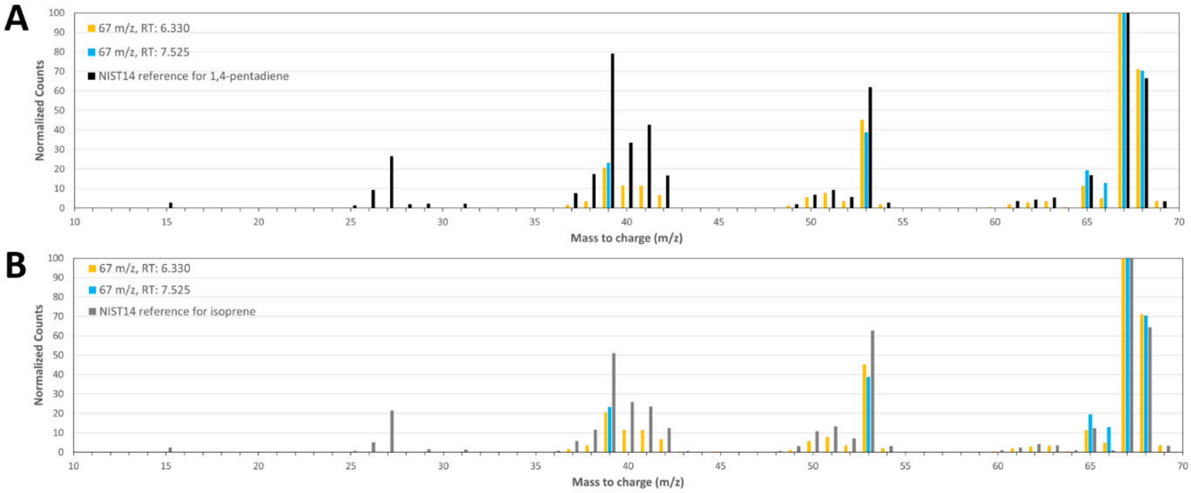

Supplemental Figure 3: Comparison of extracted chromatograms for Compound 2 (m/z 67, RT 6.330) and Compound S2 (m/z 67, RT 7.525) to the NIST14 reference for 1,4-pentadiene (**A**) and NIST14 reference for isoprene (**B**).

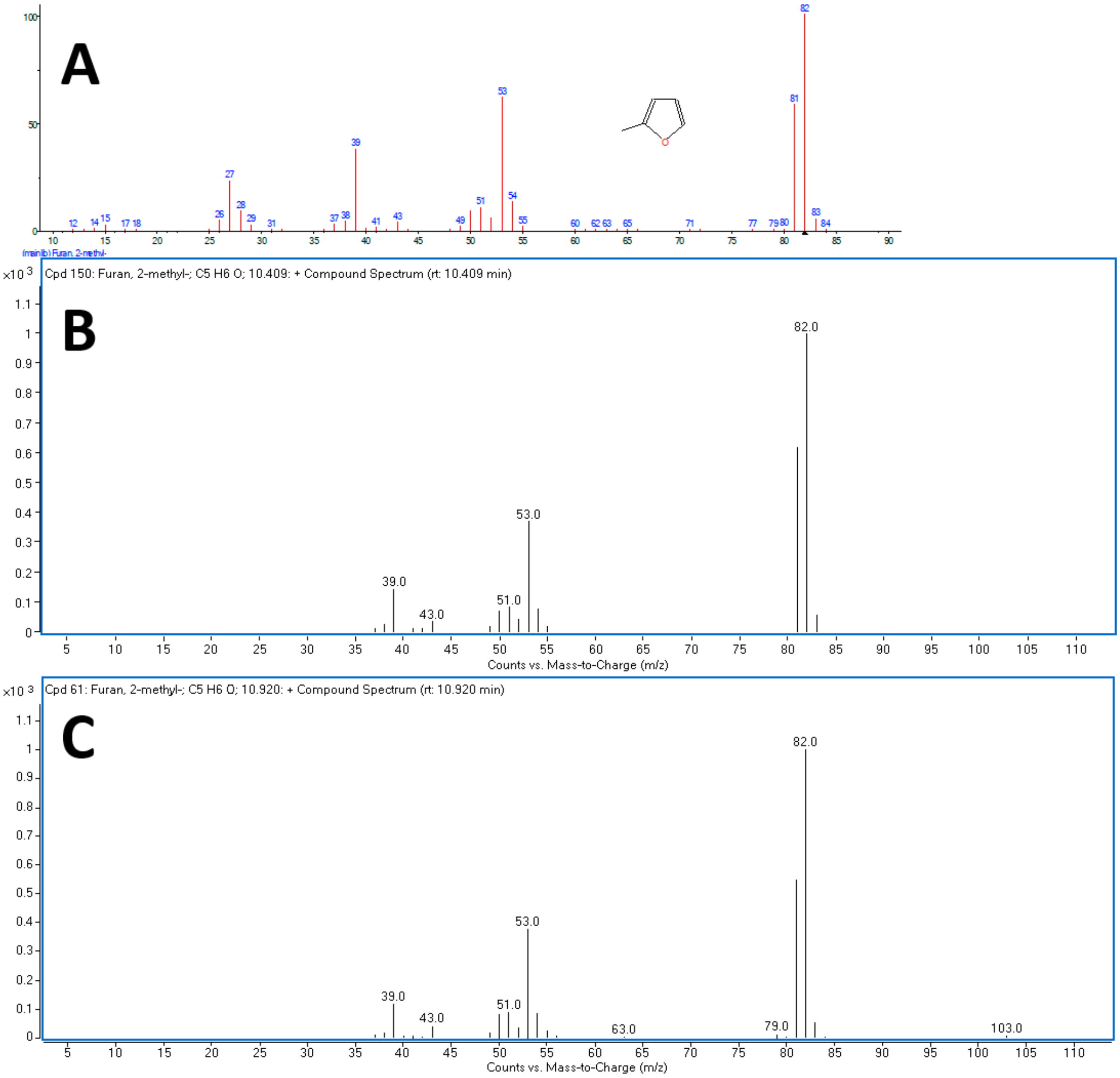

Supplemental Figure 4: NIST14 reference for 2-methylfuran (**A**) with the extracted chromatograms at 82 m/z at RT 10.409 for Compound 3 (**B**) and 82 m/z at RT 10.920 for Compound 4 (**C**).

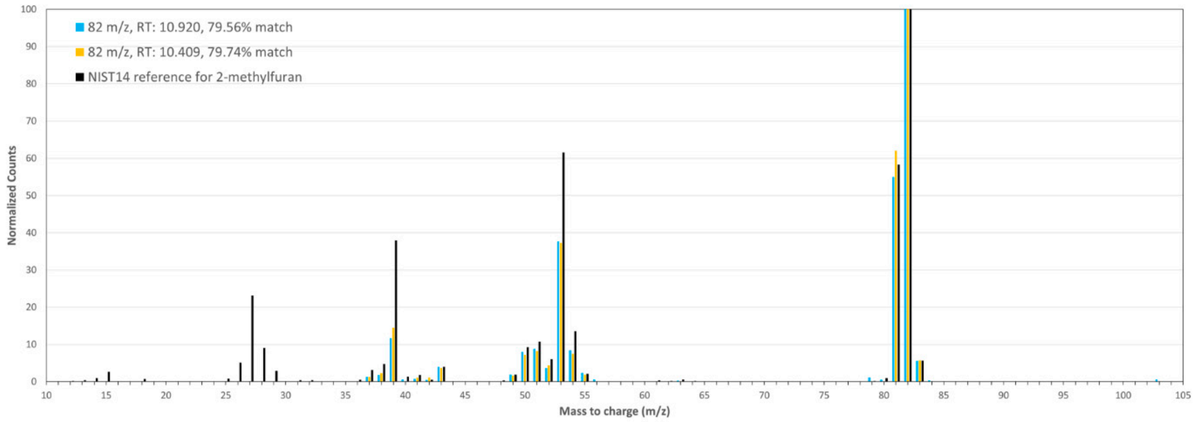

Supplemental Figure 5: Comparison of extracted chromatograms for Compound 3 at m/z 82 at RT 10.920 and Compound 4 at m/z 82 at RT 10.409 to the NIST14 reference for 2-methylfuran.
